# Castration influences intestinal microflora and induces abdominal obesity in high-fat diet-fed mice

**DOI:** 10.1038/srep23001

**Published:** 2016-03-10

**Authors:** Naoki Harada, Ryo Hanaoka, Hiroko Horiuchi, Tomoya Kitakaze, Takakazu Mitani, Hiroshi Inui, Ryoichi Yamaji

**Affiliations:** 1 Division of Applied Life Sciences, Graduate School of Life and Environmental Sciences, Osaka Prefecture University, Sakai, Osaka 5998531, Japan; 2 Interdisciplinary Graduate School of Science and Technology, Shinshu University, Kamiina, Nagano 3994598, Japan; 3 Division of Clinical Nutrition, Graduate School of Comprehensive Rehabilitation, Osaka Prefecture University, Habikino, Osaka 5830872, Japan

## Abstract

Late-onset hypogonadism (*i.e.* androgen deficiency) raises the risk for abdominal obesity in men. The mechanism for this obesity is unclear. Here, we demonstrated that hypogonadism after castration caused abdominal obesity in high-fat diet (HFD)-fed, but not in standard diet (SD)-fed, C57BL/6J mice. Furthermore, the phenotype was not induced in mice treated with antibiotics that disrupt the intestinal microflora. In HFD-fed mice, castration increased feed efficiency and decreased fecal weight per food intake. Castration also induced in an increase of visceral fat mass only in the absence of antibiotics in HFD-fed mice, whereas subcutaneous fat mass was increased by castration irrespective of antibiotics. Castration reduced the expression in the mesenteric fat of both *adipose triglyceride lipase* and *hormone-sensitive lipase* in HFD-fed mice, which was not observed in the presence of antibiotics. Castration decreased thigh muscle (*i.e.* quadriceps and hamstrings) mass, elevated fasting blood glucose levels, and increased liver triglyceride levels in a HFD-dependent manner, whereas these changes were not observed in castrated mice treated with antibiotics. The *Firmicutes*/*Bacteroidetes* ratio and *Lactobacillus* species increased in the feces of HFD-fed castrated mice. These results show that androgen (*e.g*. testosterone) deficiency can alter the intestinal microbiome and induce abdominal obesity in a diet-dependent manner.

Obesity is a global epidemic problem due to its strong association with an increased risk of cardiovascular diseases^1^,^2^. The excess accumulation of abdominal visceral fat, a diagnostic criterion of the metabolic syndrome^2^, increases the disorder in lipid metabolism, including an elevation of hepatic triglyceride levels^3^. In contrast, subcutaneous fat reduces the incidence of cardiovascular diseases, indicating the importance of body fat distribution^1^,^2^. Recent results show that the changes in intestinal microbiota are related to the development of obesity and to the increase of visceral fat mass^4^,^5^,^6^,^7^.

Testosterone is a male sexual hormone (*viz.* androgen) that exerts a broad range of male physiological functions, such as the development of reproductive organs and the emergence of sexual behaviors^8^,^9^. Hypogonadism (*i.e.* low testosterone level) increases in men the risk of obesity, cardiovascular diseases, and even mortality^10^,^11^,^12^,^13^ through the increase of body fat, in particular visceral fat^14^,^15^; and testosterone treatment reduces the amount of visceral fat^16^. Androgen deprivation therapies, such as either castration or an leteinizing hormone-releasing hormone analog for prostate cancer patients, also promote the development of obesity^17^,^18^,^19^. Because the blood bioactive testosterone level steadily drops approximately 2% per year after around the age of 20 to 30 in men^20^, the age-dependent decline of testosterone is a risk factor for the age-related prevalence of abdominal obesity and its related diseases in men^14^,^20^.

Despite increasing evidence in both clinical and epidemiological studies in humans^10^,^11^,^12^,^13^, the mechanism by which a decline of testosterone induces abdominal obesity remains largely unclear. The physiological functions of testosterone have been widely analysed in rodent castration models. Unlike humans, however, rodents lose weight after castration due to a reduction in food intake^21^,^22^ , and thus castration is considered not to cause obesity^23^.

Consumption of a high-fat diet (HFD) alters lipid metabolism^24^,^25^ and also affects the gut microbiota which are involved in the development of abdominal obesity^4^. In the present study, we investigated the interaction between a HFD and hypoandrogenism in the development of obesity in a castrated mouse model. Furthermore, we investigated the involvement of gut microbiota on the hypogonadism-induced obesity in this model.

## Results

### Effects of castration on body weight, calorie intake, feed efficiency, fecal weight, and body temperature in mice

**Figure 1:**
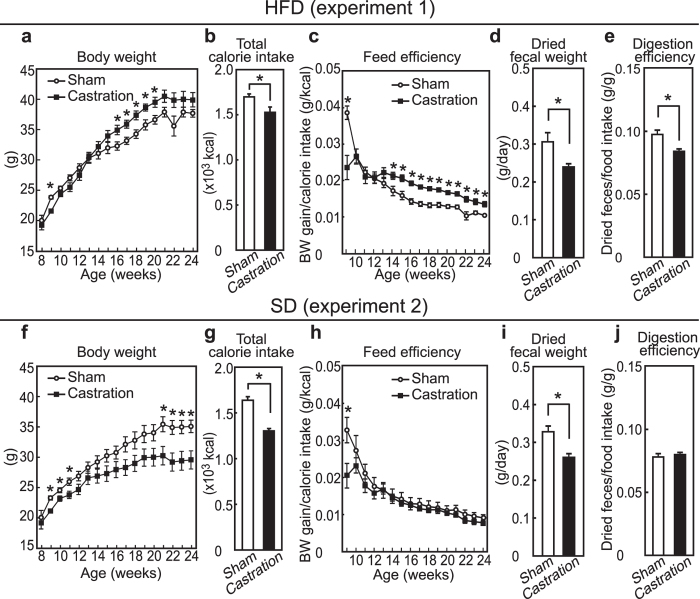
Body weight, calorie intake, feed efficiency, digestion efficiency and body temperature of sham-operated and castrated mice fed with either a SD or a HFD (experiments 1 and 2). (**a**,**f**) Growth curves for body weight; (**b**,**g**) Total calorie intake beginning after surgery at 8 weeks of age; (**c**,**h**) Calculated value of feed efficiency beginning after surgery at 8 weeks of age to the time indicated; (**d**,**i**) Dried fecal weight per day; (**e**,**j**) Digestion efficiency expressed by dividing the dried fecal weight by calorie intake. (n = 6, SD sham; n = 6, SD castration; n = 7, HFD sham; n = 7, HFD castration).

To examine the interactive effects of a HFD and hypogonadism, mice were either castrated or sham-operated at 8 weeks of age and were then fed a HFD for 16 weeks (experiment 1). After weight loss by castration, the body weight of the castrated mice exceeded that of the sham-operated mice (Fig. 1a). On the other hand, total calorie intake (*i.e.* food intake) in the castrated mice was declined to approximately 90% of that of the control mice (Fig. 1b, *p* = 0.020). Notably, castration steadily increased the cumulative feed efficiency, calculated by body weight gain divided by calorie intake, after 14 weeks of age (Fig. 1c). In contrast, castration resulted in both reduced body weight and calorie intake during the experimental period in standard diet (SD)-fed mice (experiment 2, Fig. 1f,g, *p* < 0.0001). The decrease of body weight gain by castration in SD-fed mice was due to the reduction of calorie intake because the cumulated feed efficiency was not affected by castration (Fig. 1h). Fecal output was measured as dried fecal weight at 17-weeks and was found to be decreased by castration in both HFD- and SD-fed mice (Fig. 1d, *p* = 0.018 and 1i, *p* = 0.0040). Notably, the degree of digestion, determined by a ratio of dried fecal weight-to-food intake, was decreased by castration in the HFD-fed, but was not affected in the SD-fed group (Fig. 1e, *p* = 0.0040 and 1j). These results indicate that castration-induced hypogonadism caused obesity in a HFD-dependent manner by increasing feed efficiency but not by increasing hyperphagia. In addition, metabolic changes accompanied by the decrease in fecal weight are suggested to be responsible for the elevation of feed efficiency by castration in HFD-fed mice.

### Effects of antibiotics on the castration-induced obesity in HFD-fed mice

**Figure 2:**
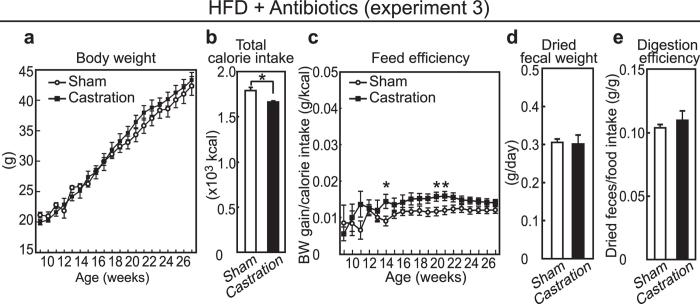
Body and nutritional parameters in HFD-fed mice given antibiotics (experiment 3). (**a**) Growth curves for body weight; (**b**) Total calorie intake beginning after surgery at 8 weeks of age ; (**c**) Calculated value of feed efficiency beginning after surgery at 8 weeks of age to the time indicated; (**d**) Dried fecal weight per day; (**e**) Digestion efficiency expressed by dividing dried fecal weight by calorie intake per day. (n = 8, sham and n = 6, castration).

We then assessed the involvement of gut microbiota in castration-induced obesity in HFD-fed mice because their derivatives account for up to half of the dried fecal weight^26^. Castrated and sham-operated mice were fed with the HFD and were given antibiotic cocktails via the drinking water to disturb gut microbiota (experiment 3). When antibiotics were provided, castration failed to cause overweight (Fig. 2a), and castrated mice had a lower calorie intake (Fig. 2b, *p* = 0.022). In mice treated with antibiotics, the castration-induced increase in feed efficiency was attenuated (Fig. 2c) and they did not exhibit the decrease in both the fecal weight (Fig. 2d) and the digestion efficiency by castration (Fig. 2e) as compared with the antibiotics-free mice.

### Effects of castration on tissue weight

The organ-to-body weight ratio in experiments 1 to 3 is summarized in Table 1. In HFD-fed mice, but not in SD-fed mice, castration increased mesenteric and perirenal/retroperitoneal adipose tissues within the visceral white adipose tissues (WATs) (*p* = 0.029 and *p* = 0.010, respectively), whereas the epididymal fat mass was not affected. The increase in visceral WATs induced by castration was not observed in castrated mice treated with antibiotics. In contrast to the changes induced in the visceral WATs by castration, the subcutaneous inguinal WATs were increased by castration in all three groups (SD, *p* = 0.04; HFD, *p* = 0.00012; HFD + antibiotics, *p* < 0.0001). Thigh muscle masses (*i.e.* quadriceps and hamstrings) were significantly decreased by castration in the HFD-fed group alone (quadriceps, *p* = 0.012; hamstrings, *p* = 0.0028), whereas castration reduced the bulbocavernosus/levator ani (BC/LA) muscle irrespective of either diets or antibiotics (all three groups, *p* < 0.0001). Castration also decreased kidney (SD, *p* = 0.00049; HFD, *p* = 0.0013; HFD + antibiotics, *p* < 0.0001) and heart (SD, *p* = 0.0043; HFD, *p* = 0.028; HFD + antibiotics, *p* = 0.0040) weight in diet- and antibiotics-independent manners, whereas castration had no effects on liver and pancreas weights.

### Effects of castration on glucose and on lipid profiles in blood, feces, and liver

The biochemical parameters in blood, feces and liver in experiments 1 to 3 are summarized in Table 2. Castration increased fasting blood glucose levels in HFD-fed mice (*p* < 0.0001), but not in SD-fed mice. By contrast, castration did not increase fasting blood glucose levels in mice fed a HFD and treated with antibiotics. Plasma insulin levels tend to be decreased by castration irrespective of diet and antibiotics, and a statistically significant difference was observed in SD-fed mice (*p* = 0.023). On the other hand, castration did not affect insulin sensitivity in all groups (Supplementary Figure S1). In HFD-fed mice, plasma free fatty acid and triglyceride levels were not affected by castration. The total cholesterol concentrations, but not the triglyceride concentrations, in feces were decreased by castration in HFD-fed mice (*p* = 0.0013), but not the SD-fed mice. The total amount, but not the concentrations, of fecal triglycerides in castrated mice fed a HFD was significantly reduced because of the reduction in fecal weight (*p* = 0.038, data not shown). Administration of antibiotics did not affect the decrease in the total cholesterol concentrations in feces (*p* = 0.00083). Interestingly, liver triglycerides were increased in castrated mice fed a HFD (*p* = 0.0024). Administration of antibiotics abnormally increased the liver triglyceride level, and castration failed to increase the level much further.

### Effects of castration on gene expression in visceral WATs and in liver

**Figure 3:**
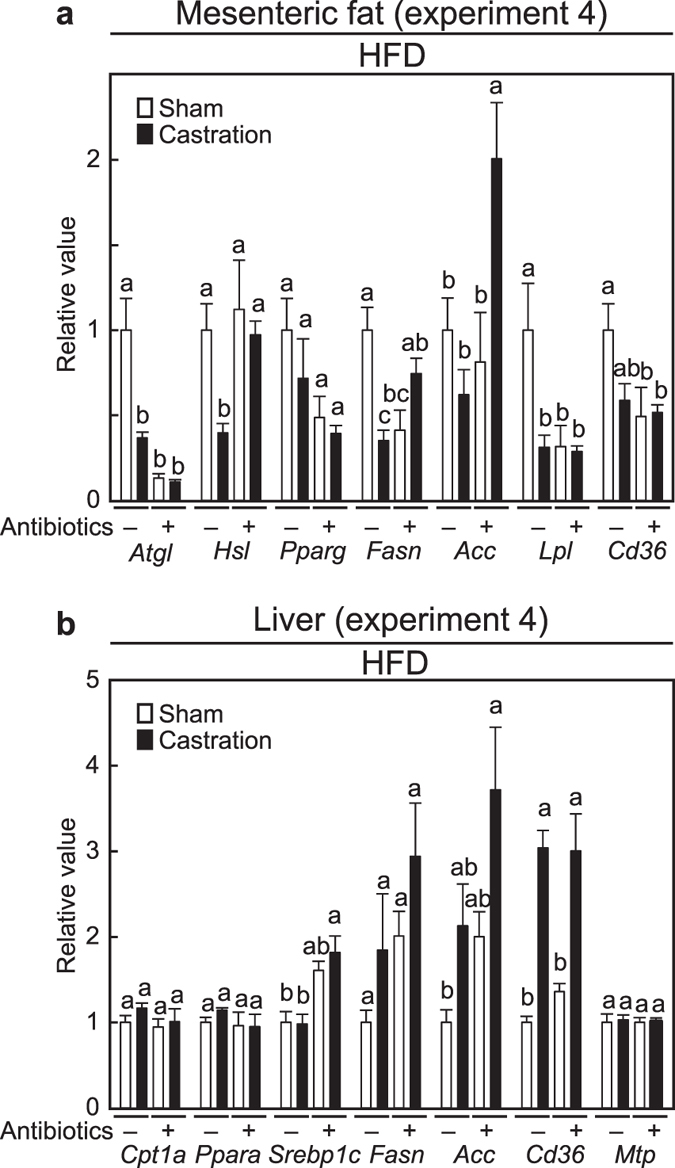
Expression gene analysis of mesenteric fat and liver at 13 weeks of age (experiment 4). (**a**) Gene expressions in mesenteric fat; (**b**) Gene expressions in the liver. (n = 7, sham without antibiotics; n = 9, castration without antibiotics; n = 6, sham with antibiotics; n = 9, castration with antibiotics).

To determine the mechanisms underlying the castration-mediated increase in the visceral WATs of the HFD-fed mice, a gene expression profile at 13 weeks was performed (experiment 4). We chose this time point because the feed efficiency was significantly different after 14 weeks of age (Fig. 1c) and the body weight gain was prominent at that time in HFD-fed mice (Fig. 1a). The effects of castration in HFD-fed mice were compared with that in HFD-fed mice plus antibiotics because the HFD is known to strongly affect lipid metabolism. The relative organ weights of the 13-weeks old mice are summarized in Supplemental Table S1. At this early stage, the increase of mesenteric and perinephric/retroperitoneal adipose tissues by castration had already occurred (*p* = 0.024 and *p* = 0.015, respectively), however, similar increases of adipose tissue were not induced in the presence of antibiotics. In the mesenteric fat of HFD-fed mice, the expressions of *adipose triglyceride lipase* (*Atgl*) and *hormone sensitive lipase* (*Hsl*), genes responsible for lipid degradation, were suppressed by castration (Fig. 3a, *p* < 0.0001 and *p* = 0.045, respectively). Castration also tended to decrease *Pparg* and *acetyl CoA carboxylase α* (*Acc*) and significantly decreased *fatty acid synthase* (*Fasn*) (*p* = 0.0004), which are lipogenic genes. *Lipoprotein lipase* (*Lpl*), which is involved in the degradation of triglyceride, was significantly decreased (*p* = 0.0078) and *Cd36*, which is involved in intake of free fatty acid (FFA), tended to be decreased by castration (*p* = 0.088). These results suggest that a decrease of lipolysis, but not an increase of either lipogenesis or FFA intake, was responsible for the increase of mesenteric fat in HFD-fed mice. Notably, in antibiotic-treated castrated mice, a decrease in either *Atgl* or *Hsl* was not detected, and the expression of *Acc* was increased in contrast to untreated castrated mice (*p* = 0.014).

Because the liver plays an important role in lipid metabolism, and castration caused triglyceride accumulation in HFD-fed mice (Table 2), we also addressed the gene expression changes in the liver at 13 weeks of age. In HFD-fed antibiotics-free mice, castration significantly increased *Cd36* (*p* = 0.0026) (Fig. 3b), suggesting an enhancement in intake of FFA. Unlike mesenteric fat, supplementation with antibiotics has almost no effect on the castration-mediated changes in hepatic gene expression.

### Effects of castration on the gut microbiota

**Figure 4:**
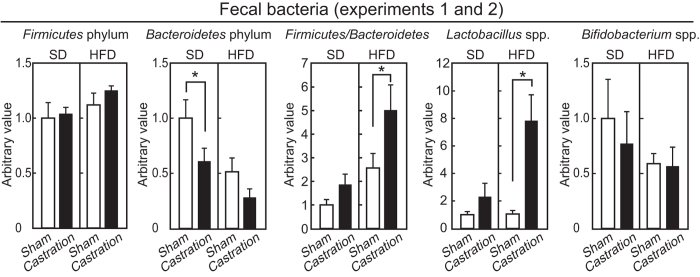
Analysis of microbiota in feces by real-time PCR (experiments 1 and 2). Relative abundances of *Firmicutes* phylum, *Bacteroidetes* phylum, *Lactobacillus* spp. and *Bifidobacterium* spp. in the feces of 13-weeks old mice (n = 6, SD sham; n = 6, SD castration; n = 6, HFD sham; n = 5, HFD castration).

To examine the effect of castration on intestinal microbiota in the SD- and HFD-fed mice (experiments 1 and 2), the genomic DNA of the microbiota were extracted from feces, and quantitative PCR was performed. In this study, we investigated the quantitative levels of the *Firmicutes* phylum, *Bacteroidetes* phylum, *Lactobacillus* species and *Bifidobacterium* species. The *Firmicutes* and *Bacteroidetes* phyla account for 80–90% of the intestinal microbacteria, and an increase in the *Firmicutes*/*Bacteroidetes* ratio is involved in obesity due to an increased capacity for energy harvest from the diet^5^. *Lactobacillus* spp. is also involved in obesity^27^,^28^. *Bifidobacterism* spp. is inversely associated with obesity^6^. As shown in Fig. 4, castration tended to increase *Firmicutes* and to decrease *Bacteroidetes* in the HFD-group, leading to a significant increase in the *Firmicutes*/*Bacteroidetes* ratio (*p* = 0.017). A marginal increase in *Firmicutes*/*Bacteroidetes* ratio was observed with castration in the SD-fed mice (*p* = 0.35). The levels of *Lactobacillus* spp. were elevated by castration in the HFD-fed mice (*p* < 0.0001), but not in the SD-fed mice (*p* = 0.34). Castration did not affect the *Bifidobacterium* spp. levels in either diet-fed group. These results indicate that castration influenced on the intestinal microbiota.

## Discussion

Low testosterone levels are associated with obesity, and androgen deprivation therapy for prostate cancer patients results in an increase in body weight accompanied by abdominal adiposity^17^,^18^,^19^,^29^. In the present study, we show that hypogonadism affected feed efficiency and caused obesity, including increased visceral fat mass, in a HFD-dependent manner in a murine castration model. Furthermore, in the presence of antibiotics, castration failed to induce an excess of visceral fat mass and obesity. Although it is still unclear whether the effects of antibiotics in reversing the effects of castration are mediated via the observed change in gut microbiota, our results offer insights suggesting that hypogonadism induces a change in gut microbiota in a diet-dependent manner.

Castration effects, such as obesity, excess visceral fat, increase of hepatic triglyceride levels, increase of fasting blood glucose levels, and decrease of fecal weight-to-food intake in HFD-fed mice were not observed when mice were treated with antibiotics. Antibiotics dramatically decreased fecal bacteria (but resistant bacteria likely remained) and obviously increased cecal weight (data not shown). Gut microbiome influence hepatic lipid metabolism and visceral fat mass, rather than subcutaneous fat mass 6, and consumption of a HFD critically alter the gut microbiota composition^30^. A recent study indicated that gut microbiota differs between males and females, and is involved in the prevalence of sex-relevant diseases^31^. We found that the *Firmicutes*/B*acteroidetes* ratio and *Lactobacillus* spp. levels were increased with castration in the HFD-fed mice. Our results support the notion that the higher *Firmicutes*/B*acteroidetes* ratio and the increased levels of *Lactobacillus* spp. are associated with the prevalence of obesity^5^,^7^,^27^,^28^.

Castration caused obesity accompanied by an increase in visceral WATs in HFD-fed mice alone. In contrast, an increase in subcutaneous WATs by castration was observed in a diet-independent manner. While AR expression is higher in visceral WATs than that in subcutaneous WATs^32^, subcutaneous and visceral WAT masses seem to be directly and indirectly regulated by androgen, respectively. This notion is supported by the fact that although increase of subcutaneous WATs was observed in castrated rats, castration did not cause an increase of visceral WAT or obesity^33^. The obese phenotypes of HFD-fed castrated mice are similar to that of the global androgen receptor knockout (AR-KO) mice exhibiting a late-onset obesity^34^,^35^. Among the five developed global AR-KO mice, including deletion of its DNA-binding domain, only two lineages (constructed by Kato’s group and by Chang’s group) significantly exhibit late-onset obesity accompanying the accumulation of visceral fat^34^,^35^,^36^,^37^,^38^, whereas the other three lineages did not exhibit the phenotype. Our results raise the possibility that these inconsistent results are derived from the difference in intestinal microflora due to the different genomic backgrounds of the mice and the type of food they were fed because these factors strongly affect intestinal microflora^4^,^39^. In addition, HFD-dependent obesity is observed in liver-specific AR-KO mice that accumulate triglyceride in the liver and exhibit visceral obesity^40^. In this study, triglyceride accumulation in the liver was also observed in HFD-fed castrated mice. These results suggest that castration-induced dyslipidemia in the liver is also involved in increased visceral fat mass and in low androgen-induced obesity.

Obesity in castrated mice fed a HFD was caused by the elevation of feed efficiency but not by hyperphagia. In general, castration and ovariectomy cause hypophagia and hyperphagia, and result in lower and greater weight gain in male and in female rodents, respectively^21^,^41^. Therefore, the mechanism underlying the induction of obesity by castration in HFD-fed mice was different from that in the obese female mice induced by ovariectomy^41^. The obese AR-KO male mice show reduced physical activity accompanied by a reduction in O_2_ consumption^35^. In HFD-fed mice, castration increased the *Firmicutes*/*Bacteroidetes* ratio that has been shown to increase the capacity for harvesting energy from diet^5^, and that decreased fecal weight-to-food intake in our study. Taken together, these results suggest that both a decrease of energy expenditure and an increase in energy harvested from diet are involved in the elevation of feed efficiency induced by hypogonadism.

The molecular mechanism responsible for the accumulation of visceral fat seems to rely on a decrease in lipolysis and not on an increase in lipogenesis. *Atgl* and *Hsl* in mesenteric fat were suppressed by castration in HFD-fed mice, but remained unchanged by antibiotic treatment. Substantial decreases of *Hsl* in intraperitoneal WATs are observed in global AR-KO obese mice^42^. These results indicate that decreased lipolysis in visceral WATs is similar in the AR-KO mice and in the castration HFD-fed murine model of the obese phenotype. Conversely, in the liver of HFD-fed castrated mice, triglyceride accumulated presumably through increasing lipogenesis (*i.e.* increases of *Fasn* and *Acc*) and FFA intake (*i.e.* increase of *Cd36*). These changes in the liver were unaffected by antibiotic treatment and are similar to that in the liver-specific AR-KO mice (*i.e. Srebp-1c, Acc, Scd1* and *Pparg*) that are obese^40^.

A small thigh circumference increases the risk of both heart disease and mortality^43^. Low testosterone levels are also risks factors for cardiovascular disease and for a shortened life-span^10^,^11^,^12^. Our results show that castration caused the loss of quadriceps and hamstrings muscle masses leading to a decreased thigh circumference, all occurring in a HFD-dependent manner. Therefore, a decreased thigh circumference might be involved in the relationship between low testosterone levels and cardiovascular disease or a shortened life-span. The results from global AR-KO, muscle-specific AR-KO, and castration mouse models on leg and thigh muscle mass are inconsistent^22^,^42^,^44^,^45^,^46^, and in this study, thigh muscle mass was not decreased by castration when mice were given antibiotics. In addition, our results suggest that androgen deprivation causes sarcopenic obesity in a HFD-dependent manner.

Castration increased fasting blood glucose levels in the HFD-fed mice, whereas castration did not affect insulin sensitivity even in the HFD-fed mice. One of two lineage of AR-KO mice having an obese phenotype exhibits a decrease in insulin sensitivity^34^; whereas the other lineage shows no effect on insulin sensitivity^33^,^42^. In this study, insulin sensitivity (after a 6 h fast) was unchanged, but fasting glucose (after a 15 h fast) was increased with castration in the HFD-fed mice. On the other hand, the basal plasma insulin levels (after a 4 h fast) slightly decreased with castration in the HFD-fed mice (*p* = 0.27, Table 2). Our results may suggest that insulin secretion was impaired by castration in the HFD-fed mice. This possibility is supported by published manuscripts suggesting that testosterone is involved in β-cell function^47^,^48^.

AR-KO mice models are beneficial for analysing the effects of androgens. However, global AR-KO male mice are born with female-like external genitalia and small, undescended testes^36^, casting some doubt that the phenotypes of AR-KO mice entirely represent that of the age-related decline of testosterone. In the present study, we used C57BL/6J mice, which is a widely used inbred strain for constructing genetically modified mice. We conclude that a diet- and castration-induced obesity model will be a powerful tool for the study of hypogonadism-related diseases.

## Methods

### Animals

C57BL/6J mice (7 weeks old) were obtained from Kiwa Laboratories (Wakayama, Japan). C57BL/6J mice are sensitive to high-fat diet-induced obesity and have glucose intolerance^49^. Mice were housed individually in a room with a 12:12 dark/light cycle (light period starting from 8:00) and with controlled temperature (23 ± 2 °C) and humidity (60 ± 10%). Mice were allowed free access to diet and to water. At 8 weeks of age, mice were castrated or sham-operated under anesthesia. Then, their food was changed to either SD or HFD from standard chow (CE-2, CREA Japan, Tokyo, Japan). Feces were collected at 13 (for analysing microbiota) and 17 (for analysing digestion efficiency) weeks of age for a week, and fecal weight was measured after freeze-drying. The digestion efficiency at 17 weeks was calculated by dividing the total dried fecal weight-to-calorie intake. Mice were sacrificed at either 24 (experiments 1 and 2), 27 (experiment 3) or 13 (experiment 4) weeks of age under anesthesia after 4 hours of fasting, and tissue weights were measured. Fasting blood glucose levels were measured using OneTouch Ultra (Lifescan; Johnson & Johnson, Milpitas, CA, USA) after 15 hours of fasting at 21 weeks. The insulin tolerance test was performed by injecting 1 U/kg human insulin after a 6 h fast at 22 weeks. All mice were individually housed (n = 6–9). Animal experiments were approved by the Animal Care and Use Committee of Osaka Prefecture University and were performed in compliance with its guidelines.

### Diets

The compositions of SD and HFD are listed in Supplemental Table S2. Casein, cornstarch, α-cornstarch, corn oil, lard, beef tallow, cellulose, mineral mixture, and a vitamin mixture were obtained from CLEA Japan. Ingredients were mixed, and powdered diets were stored at −20 °C until use. Fresh food was given to the animals 3 times per week. Antibiotics (1 g/L of sodium ampicillin, 0.5 g/L of vancomycin hydrochloride, 1 g/L of neomycin sulfate and 1 g/L of metronidazole) were administrated through the drinking water after castration.

### Measurement of insulin, triglyceride, FFA, and cholesterol levels

Plasma insulin levels were determined using an insulin ELISA kit (Shibayagi, Gunma, Japan). Lipids were extracted from both the liver and feces by Folch’s method^50^. Triglyceride levels in the liver, the plasma, and feces were determined with the triglyceride E-Test (Wako, Osaka, Japan). Plasma, fecal, and liver cholesterol levels were determined with the cholesterol E-Test (Wako).

### Real-time PCR

Total RNA was isolated from tissues, and cDNA was synthesized using ReverTra Ace (TOYOBO, Osaka Japan) and dT20 primers. Genomic DNA was extracted from fecal samples using zirconia beads (*ϕ* 5 mm and *ϕ* 0.2 mm) with a multi-beads shocker (MB755U(S), Yasui Kikai, Osaka, Japan) and the QIAamp Fast DNA Stool Mini Kit (Qiagen, Hilden, Germany). Quantitative RT-PCR was performed using SYBR Premix Ex *Taq* II (TAKARA Bio, Shiga, Japan) with the various sets of primers (Supplemental Table S3). The PCR program was 95 °C, 60 sec followed by 40 cycles (2-steps: 95 °C, 30 sec; annealing and elongation, 30 sec or 3 steps; 95 °C, 30 sec; annealing, 30 sec; elongation, 72 °C, 30 sec). The relative expression of target genes was calculated using the Ct value fitted to a standard curve which was obtained from a series of diluted cDNA, and normalized by that of the control gene (*e.g.* β-actin or 16 S rRNA for total bacteria).

### Statistical analysis

Data were analysed by the Student’s *t*-test or one-way analysis of variance followed by Tukey-Kramer’s post-hoc testing using the JMP statistical software version 8.0.1 (SAS Institute, Cary, NC, USA). Data are shown as means ± SEM, and a statistically difference between groups is considered when *p* < 0.05. Experiments 1 to 4 were independently performed. Data were compared within the same experiment only.

## Additional Information

**How to cite this article**: Harada, N. *et al*. Castration influences intestinal microflora and induces abdominal obesity in high-fat diet-fed mice. *Sci. Rep.* **6**, 23001; doi: 10.1038/srep23001 (2016).

## Supplementary Material

Supplementary Information

## Figures and Tables

**Table 1:** Relative organs weights (% of body weight) (experiments 1–3).

	SD		HFD		HFD + Antibiotics	
	Sham	Castration	Sham	Castration	Sham	Castration
Visceral WAT						
Mesenteric	1.75 ± 0.10	1.62 ± 0.17	2.00 ± 0.22	2.73 ± 0.18*	2.24 ± 0.23	2.46 ± 0.30
Perirenal/retroperitoneal	1.71 ± 0.15	1.75 ± 0.16	2.07 ± 0.16	2.88 ± 0.21*	2.64 ± 0.13	2.63 ± 0.21
Epididymal	3.73 ± 0.37	3.31 ± 0.72	4.65 ± 0.30	4.62 ± 0.47	4.78 ± 0.35	5.58 ± 0.74
Subcutaneous WAT						
Inguinal	2.50 ± 0.21	3.31 ± 0.28*	4.91 ± 0.27	7.87 ± 0.46*	3.95 ± 0.21	5.55 ± 0.41*
Skeletal muscle						
Quadriceps	1.00 ± 0.04	1.03 ± 0.06	0.85 ± 0.04	0.69 ± 0.04*	1.18 ± 0.03	1.11 ± 0.09
Hamstrings	2.09 ± 0.04	2.13 ± 0.06	1.93 ± 0.04	1.60 ± 0.04*	2.31 ± 0.08	2.10 ± 0.20
BC/LA	0.26 ± 0.01	0.04 ± 0.003*	0.23 ± 0.009	0.02 ± 0.002*	0.27 ± 0.01	0.03 ± 0.004*
Others						
Liver	4.19 ± 0.16	3.80 ± 0.12	4.15 ± 0.26	4.00 ± 0.23	3.64 ± 0.07	3.75 ± 0.49
Kidney	1.30 ± 0.05	0.99 ± 0.03*	1.30 ± 0.10	0.85 ± 0.03*	1.33 ± 0.04	0.91 ± 0.03*
Pancreas	0.83 ± 0.02	0.89 ± 0.09	0.88 ± 0.08	0.74 ± 0.06	0.77 ± 0.03	0.79 ± 0.04
Heart	0.41 ± 0.01	0.37 ± 0.01*	0.37 ± 0.01	0.32 ± 0.01*	0.35 ± 0.01	0.30 ± 0.01*

Data are expressed as means ± SEM. Statistical differences (*p < 0.05) were determined between sham-operated mice and castrated mice.

**Table 2:** Biochemical parameter in blood, plasma, feces and liver (experiments 1–3).

	SD		HFD		HFD + Antibiotics	
	Sham	Castration	Sham	Castration	Sham	Castration
Blood						
Glucose (mg/dl)	82.5 ± 5.0	94.7 ± 5.8	94.1 ± 3.6	133.7 ± 6.0*	86.7 ± 4.8	99.6 ± 7.3
Plasma						
Insulin (ng/ml)	1.4 ± 0.3	0.5 ± 0.2*	2.5 ± 0.5	1.8 ± 0.3	1.5 ± 0.5	1.0 ± 0.2
Free fatty acid (mM)	1.33 ± 0.09	1.33 ± 0.10	1.04 ± 0.06	1.03 ± 0.05	4.39 ± 0.19	3.92 ± 0.16
Triglyceride (mg/dl)	135 ± 14	86 ± 10*	79 ± 11	70 ± 9	52 ± 7.0	49 ± 4.0
Feces						
Triglyceride (mg/g)	10.7 ± 0.4	10.2 ± 0.5	11.2 ± 0.4	10.7 ± 0.4	5.4 ± 1.0	5.3 ± 0.7
Total Cholesterol (mg/g)	5.2 ± 0.6	5.3 ± 0.2	6.8 ± 0.7	3.7 ± 0.2*	4.9 ± 0.4	3.4 ± 0.3*
Liver						
Triglyceride (mg/g)	30.0 ± 3.9	39.2 ± 9.0	49.1 ± 5.9	92.3 ± 9.6*	151 ± 29	177 ± 10
Total Cholesterol (mg/g)	4.4 ± 0.3	5.3 ± 0.6	6.0 ± 0.3	6.4 ± 0.3	13.7 ± 1.2	14.2 ± 0.5

Data are expressed as means ± SEM. Statistical differences (*p < 0.05) were determined between sham-operated mice and castrated mice. Blood glucose levels and feces were analyzed at 21 and 17 weeks, respectively. Plasma and liver were analyzed after dissection.
