# Supplementary material for: Castration influences intestinal microflora and induces abdominal obesity in high-fat diet-fed mice
Source: Sci Rep. 2016 Mar 10;6:23001. doi: 10.1038/srep23001 (PMC4785334; doi:10.1038/srep23001)
Supplement: Supplementary Information [file srep23001-s1.pdf]

Castration influences intestinal microflora and induces abdominal obesity in high-fat diet-fed mice

Naoki Harada, Ryo Hanaoka, Hiroko Horiuchi, Tomoya Kitakaze, Takakazu Mitani, Hiroshi Inui, and Ryoichi Yamaji.

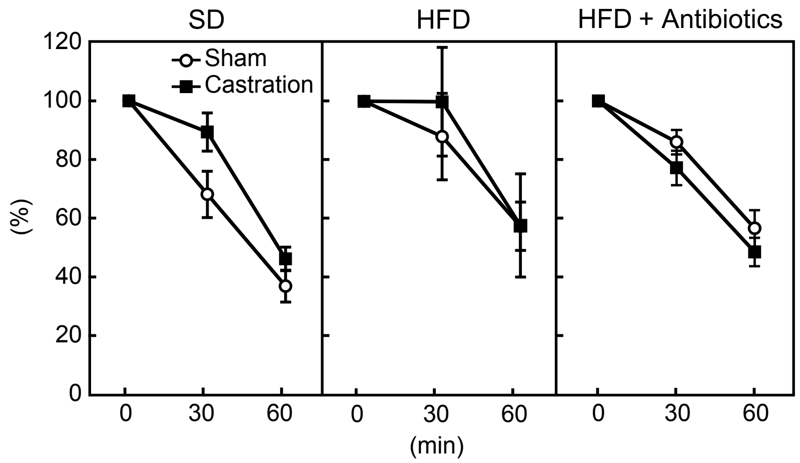

Supplelmentary Figure S1. Insulin torelance test (experiments 1‒3). Mice at 22-weeks old were fasted for 6 h and human insulin (1 U/kg) were intraperitoneally injected. Blood glucose levels were measured and expressed as percentage change from baseline (0 min). (n=6, SD sham or castration; n=7, HFD without antibiotics sham or castration; n=9, HFD with antibiotics sham; n=6, HFD with antibiotics castration)

Supplementary Table S1. Relative weight of organs (% of body weight) of 13-week old mice (experiment 4)

	HFD		HFD+Antibiotics	
	Sham	Castration	Sham	Castration
Visceral WAT				
Mesenteric	0.65±0.11[b]	1.14±0.11[a]	0.57±0.07[b]	0.55±0.10[b]
Perirenal/retroperitoneal	0.54±0.11[b]	1.07±0.11[a]	0.51±0.07[b]	0.45±0.11[b]
Epididymal	1.49±0.21[a]	1.56±0.16[a]	1.18±0.17[a]	1.09±0.13[a]
Subcutaneous WAT				
Inguinal	1.33±0.12[b]	2.23±0.16[a]	1.23±0.12[b]	1.26±0.22[b]
Skeletal muscle				
Quadriceps	1.43±0.05[a]	1.22±0.06[a]	1.35±0.08[a]	1.37±0.04[a]
Hamstrings	3.03±0.18[a]	2.86±0.05[a]	3.00±0.08[a]	2.78±0.12[a]

Data are expressed as means ± SEM. Data were analysed by one-way ANOVA followed by Tukey-Kramer's post-hoc test. Different letters mean statistical differences (p<0.05).

Supplementary Table S2. Composition of the diets (experiments 1–4)

	SD	HFD
Ingredients (g/kg)		
Casein	200.00	200.00
L−Cystine	3.00	3.00
Cornstarch	397.44	167.44
α−Cornstarch	132.00	132.00
Sucrose	100.00	100.00
Corn oil	70.00	20.00
Lard	0.00	1400.00
Beef tallow	0.00	1400.00
Cellulose	50.00	50.00
Mineral Mix (AIN−93G)	35.00	35.00
Vitamin Mix (AIN−93)	10.00	10.00
Choline chloride	2.50	2.50
Tert-butylhydroquinone	0.06	0.06
Energy density (kcal/g diet)	3.69	4.82
Macronutrients (% total energy)		
Protein	19%	15%
Carbohydrate	62%	33%
Fat	19%	52%

Supplementary Table S3. Primers for amplification of genes by qRT-PCR

Gene	Sequences of primers	Anneal Temp (°C)	Steps
mouse *Acc*	5′-GGAGATGTACGCTGACCGAGAA-3′<br>5′-ACCCGACGCATGGTTTTCA-3′	60	2
mouse *Atgl*	5′-TGTGGCCTCATTCCTCCTAC-3′<br>5′-TCGTGGATGTTGGTGGAGCT-3′	58	2
mouse *Cd36*	5′-GCCAAGCTATTGCGACATGA-3′<br>5′-CAATGGTTGTCTGGATTCTGG-3′	58	2
mouse *Cpt1a*	5′-GATCTACAATTCCCCTCTGCTCT-3′<br>5′-TAGAGCCAGACCTTGAAGTAACG-3′	60	2
mouse *Fasn*	5′-TGGGTTCTAGCCAGCAGAGT-3′<br>5′-ACCACCAGAGACCGTTATGC-3′	60	2
mouse *Hsl*	5′-GCTGGGCTGTCAAGCACTGT-3′<br>5′-GTAACTGGGTAGGCTTGCCAT-3′	58	2
mouse *Lpl*	5′-GTGGCCGAGAGCGAGAACAT-3′<br>5′-GCTTTCACTCGGATCCTCTC-3′	60	2
mouse *Mtp*	5′-CATCTCCACAGTGCAGTTCTCACA-3′<br>5′-GGAGTTCACATCCGGCCACTA-3′	58	2
mouse *Pparg*	5′-GGAAGCCCTTTGGTGACTTTATGG-3′<br>5′-GCAGCAGGTTTCTTGGATGTC-3′	58	2
mouse *Ppara*	5′-AATGCAATTCGCTTTGGAAG-3′<br>5′-GGCCTTGACCTTGTTCATGT-3′	60	2
mouse *Srebp1c*	5′-AGCTGTCGGGGTAGCGTCTG-3′<br>5′-GAGAGTTGGCACCTGGGCTG-3′	55	2
mouse *β-Actin*	5′-TTGCTGACAGGATGCAGAAG-3′<br>5′-GTACTTGCGCTCAGGAGGAG-3′	60	2
Bacteroidetes phylum	5′-GGAGYATGTGGTTTAATTCGAAGCA-3′<br>5'-AGCTGACGACAACCATGCAG-3'	60	3
Firmicutes phylum	5′-GGARCATGTGGTTTAATTCGATGAT-3′<br>5′-AGCTGACGACAACCATGCAC-3′	60	3
Bifidobacterium spp.	5′-TCGCGTCYGGTGTGAAAG-3′<br>5′-CCACATCCAGCRTCCAC-3′	60	3
Lactobacillus spp.	5′-AGCAGTAGGGAATCTTCCA-3′<br>5′-CACCGCTACACATGGAG-3′	60	3
All bacteria	5′-GTGSTGCAYGGYTGTCGTCA-3′<br>5′-ACGTCRTCCMCACCTTCCTC-3′	60	3
